# Predictors of psychological stress occurring after the first wave of the COVID-19 pandemic in Poland: A cross-sectional study

**DOI:** 10.3389/fpsyt.2022.1102728

**Published:** 2023-01-10

**Authors:** Piotr Długosz

**Affiliations:** Pedagogical University of Kraków, Kraków, Poland

**Keywords:** COVID-19 pandemic, psychological distress, predictors of stress, neuroticism, Poland

## Abstract

The article presents the results of research aimed to identify the predictors of psychological distress among Poles 7 months after the occurrence of the first case of COVID-19. In order to gather the research material, the CAWI on-line survey method was applied and carried out within the framework of the Ariadna Research Panel on the sample of 1,079 Poles aged 15 and over. The results of the conducted research indicate that Polish society experienced psychological distress during the first wave of the pandemic. According to the Kessler Psychological Distress Scale (K10), no mental disorders were observed among 36% of Poles, mild mental disorders were observed among 23% of respondents, average levels of disorders were observed among 18% of respondents, whereas high levels of disorders were observed among 23% of respondents. A hierarchical linear regression analysis was used to identify the risk factors of psychological distress. In the first stage, socio-demographic variables explained 13% of the distress variance. In the second stage, the variables measuring social nuisances of the pandemic were introduced, which increased the percentage of the explained stress variance to 24%. In the third stage, the introduced psychological variables increased the percentage of the explained variance to 65%. The main factor which increased stress levels was neuroticism. The conducted analyses have shown that the lack of social, economic and psychological capital significantly increases the susceptibility to distress when a threat to life and health lasts for a prolonged period of time.

## 1. Introduction

On the 4th of March 2020, the first case of coronavirus in Poland was confirmed in the Lubusz Voivodeship, which borders Germany. The person who contracted coronavirus arrived in Poland from Germany by bus. One week later, the Polish Ministry of Health confirmed the first infection in a child and the first fatalities. A month later, 3,383 cases and 71 COVID-19 fatalities were confirmed. The first wave of the pandemic in Poland was benign. Until July 2020, there were approximately 300 confirmed cases a day, then 700 new cases a day in August and more than 800 cases a day in September were confirmed. The second wave started in October and the number of reported incidences increased to 9,000 cases a day (1).

Until October, there were approximately 90,000 cases of coronavirus in Poland. At the same time, there were 290,000 cases in Germany, 550,000 cases in France, 758,000 cases in Spain, 313,000 cases in Italy, 167,000 cases in Russia and 203,000 cases in Ukraine. Poland was among countries which were undergoing the pandemic quite safely (2). This is confirmed by the data which shows the number of confirmed cases among 100,000 inhabitants. At the end of the first wave, this index showed 246 people in Poland, 355 people in Germany, 862 people in France, 697 people in the Czech Republic and 917 people in Sweden (3).

The pandemic has triggered an array of emotional, physical, and economic issues. COVID-19 has already led to diverse mental health problems, including anxiety, depression and post-traumatic stress disorder (4–8). On the basis of a meta-analysis, it has been determined that symptoms of anxiety (6.33–50.9%), depression (14.6–48.3%), post-traumatic stress disorder (7–53.8%), psychological distress (34.43–38%), and stress (8.1–81.9%) were observed in many countries (9).

The conducted comparative analyses of mental health indices, measured before and during the pandemic, indicate the deterioration of mental health in many countries (10–13). Apart from a threat to life and health among the society, nuisances connected with the quarantine, social isolation, deprivation of needs, the loss of job and financial resources and new stressors have emerged during the development of the pandemic. The unpredictable nature of the virus creates circumstances of ongoing stress, which can increase the risk of people developing psychological disorders (14).

The pandemic of COVID-19 is severely affecting mental health worldwide. Considerable knowledge about the influence of the pandemic on mental health has already been acquired (15, 16). Risk factors having influence on the level of experienced psychological distress have been studied to a lesser extent. Numerous research conducted on representative samples indicates that there are individual and group differences in the susceptibility to stress related to the pandemic.

On the basis of the conducted analyses, three groups of predictors having influence on the level of stress experienced during the pandemic have been distinguished. The first category includes socio-demographic variables, such as sex, age, marital status, financial standing, education level and social connections (17–20). The second category of risk factors includes social effects of the pandemic, such as a decrease in social security, the sense of uncertainty, the sense of deprivation and a change in one’s lifestyle (21, 22). The third category of variables includes individual psychological traits, such as neuroticism, stability, optimism and internalization of control (18, 23, 24).

The studies into the risk factors of stress conducted to date rarely consider the question of influence of contextual factors (25). The impact that contextual factors have on stress is well-expressed in the Conservation of Resources (COR) stress theory (26–28) and the theory of life change (29).

Therefore, in the following stage, an attempt was taken to verify whether the loss of resources connected with the lockdown is indicated by a change in one’s lifestyle, deprivation of needs and limitations of social functioning which results from the pandemic, and whether they will be significant, similarly to demographic or psychological factors.

The main aim of this paper is to demonstrate the predictors of stress and their impact on mental health after the first wave of the pandemic, illustrated by the example of Poland.

The study of stress and its predictors fits into findings on psychological effects of disasters (30, 31). Thanks to the acquired knowledge, it will be possible to get to know better the functioning of the society in the situation of a crisis, and develop solutions which could allow for helping people with their return to a good mental wellbeing.

It is assumed that, apart from psychological and socio-demographic factors, the factors connected with the loss of social and economic resources will have a crucial role in the increase of stress during the Covid-19 pandemic.

## 2. Materials and methods

### 2.1. Participants and procedure

The study was conducted on a representative nationwide Polish sample (*N* = 1,079; 554 women; age range = 15–94, M = 42.4, SD = 16.7). The participants were recruited using the Ariadna Polish on-line panel, which has over 110,000 active panel members, aged 15 and over. The survey method with the use of the (CAWI) Computer-Assisted Web Interview (CAWI).

The selection of the research sample was carried out in two stages. In the first stage, the population was segregated into subgroups based on mutual exclusivity. Then, from such separated groups, respondents were selected based on the stratification model, including gender, age, place of residence and region, and level of education. In the second stage, the respondents were recruited based on demographic data from the Central Statistical Office in Poland.

Each email contained a unique link to the study that worked only once and only for the particular panel member. When the participant clicked on the link, they were transferred to ARIADNA’s research platform and, after reading the information about the study and giving informed consent, the participant started the study.

All procedures were conducted in accordance with the ethical standards of the institutional and/or Polish national research committees, and with the 1964 Helsinki declaration and its later amendments or comparable ethical standards.

This method was used due to social isolation, which is taking place during the pandemic. Nonetheless, as shown by the results of research, the questions related to the measurement of mental health indicators conducted with the use of an on-line survey are as accurate as standard paper-and-pencil tests (32, 33).

The studies were carried out between the 26th and the 30th of October 2020. On the day of the commencement of the research, there were 1,584 cases of COVID-19 in Poland, whereas on the 30th of September there were 1,552 cases. The research was conducted a day before the second wave of the pandemic, as from October the number of incidences increased dramatically and by the end of the month there were over 22,000 cases. Therefore, the research was conducted in the circumstances of a relative stabilization of the number of incidences after the summer break. It may be assumed that it was a perfect moment to capture the consequences of the pandemic after the first wave.

### 2.2. Demographic characteristics

The research form included questions regarding the following demographic factors: gender, age, education, socio-economic status, place of residence, religiousness (Table 1).

**TABLE 1:** Frequency (*N*) and percentage (%) of answers to demographic questions.

		*N*	%
Gender	Male	525	49
	Female	554	51
Age	15–24	211	20
	25–34	202	19
	35–44	171	16
	45–54	185	17
	>55	310	29
Education	Below-secondary	180	17
	Secondary	475	44
	Higher	424	39
Evaluation of financial status	Bad	154	14
	Average	343	32
	Good	582	54
Place of residence	Village	399	37
	<20,000	141	13
	20,000–99,000	219	20
	100,000–500,000	189	18
	>500,000	131	12
Religiosity	Very religious	98	9
	Religious	592	55
	Undecided	212	20
	Non-religious	177	16

#### 2.2.1. Social capital index

Social capital is a significant factor which is of a strategic importance in the situation of stress (Table 2). It provides an individual with the necessary social support (34, 35). There is evidence of its positive impact on the psychological wellbeing of the society (36). In the measurement of social capital, five items were used for asking about the possessed support, with the use of a nominal “yes, no, I don’t know” scale. The distribution of the social capital in the researched group is presented below. Scores range from 0 to 5. The average social capital index was 3.4, (SD = 1.6).

**TABLE 2:** Frequency (*N*) and percentage (%) social capital.

	*N*	%
Wanted to find a good job	523	49
Found themselves in a difficult situation and needed money	753	70
Got sick and needed care	829	77
Needed to handle official/administrative matters	797	74
Needed help with explaining a complicated case	772	72

### 2.3. Measurement of social effects of the pandemic

#### 2.3.1. Loss of economic resources index

The impact of lockdown on the economic status of Poles was researched in-depth as well. To this end, the loss of economic resources index was used. It was made of four yes/no answers to the question if during the pandemic the researched individual experienced the loss of their job, a decrease in the number of working hours, taking up remote work, focusing on looking after their children at home. The changes on the labor market were most often based on taking up remote work (15%), a decrease in one’s working hours (14%), the loss of job (6%) and focusing on looking after children. Scores ranged from 0 to 4. The higher the score, the bigger the loss of economic resources. The average on the scale was 0.4, SD = 0.5.

#### 2.3.2. Life position decrease index

In order to verify the extent to which the coronavirus epidemic undermined the life position of the respondents, the Cantril Scale (CS) (37) was used to ask about the experienced life position on the scale ranging from 1 to 10 (where 1 is the worst possible life and 10 is the best possible life). The respondents were asked to compare the position they had before the pandemic and the position they have during the quarantine. The average result of the life position before COVID-19 was 6.92, SD = 1.8, whereas during the quarantine the result was 6.47, SD = 1.9. The life position decrease index was made on the basis of the calculation of the difference between the position estimated on the day of the research and the position before the pandemic. Scores on this index range from -9 to 9. Higher results mean a bigger decrease in one’s life position. The average on the scale was 0.4, SD = 1.6. When presented as a percentage, it means that 53% of respondents have not experienced a decrease in the standard of living, 13% have experienced its increase, whereas 34% of respondents have experienced a decrease in the standard of living.

#### 2.3.3. Life changes index

The quarantine has forced individuals to change their social practices. After social distancing, lockdown and other restrictions have been imposed, part of Poles had to give up their then-current activities. A change of one’s habits may be a serious source of stress, as indicated by research (29). The life changes index was constructed on the basis of answers to four questions regarding the fact whether the pandemic had impact on cancelation of vacation, avoidance of social and family meetings, participation in cultural events and religious services. The gathered responses indicate that 45% of respondents resigned from a tourist trip, 39% resigned from social and family meetings, 56% of respondents resigned from their participation in cultural events and 32% of respondents resigned from religious services. Scores ranged from 0 to 4. The higher the score, the higher the level of life changes. The average on the scale was 1.7, SD = 1.2.

#### 2.3.4. Deprivation of needs index

Apart from a change in social habits, the pandemic has significantly influenced the possibility of satisfying different basic and higher-level needs. The closing-up of shops, shopping malls, fitness centers, workplaces and national borders has resulted in serious restrictions as regards satisfying one’s needs. Inability to use social infrastructure and limitations in spending one’s free time, entertainment and participation in cultural events may generate frustration and have negative impact on mental health of Poles.

The deprivation of needs index has been prepared on the basis of answers pertaining to the impact of the COVID-19 pandemic on various aspects of social functioning. The instrument measuring the deprivation of needs includes eight questions in which respondents marked whether the pandemic had a positive, negative or no impact on a particular aspect of living. Detailed distribution of answers shown in percentage is presented in Table 3.

**TABLE 3:** The influence of pandemic on the possibility to fulfill one’s needs.

	Decreased		Not changed		Increased	
	***N***	**%**	***N***	**%**	***N***	**%**
Possibility to fulfill one’s food and nutritional needs	110	10	911	84	58	6
Possibility to spend time with their family	205	19	718	67	156	14
Fulfilling one’s professional duties	187	17	834	77	58	5
Financial standing	283	26	740	69	56	5
Cultural needs	430	40	587	54	62	6
Their own or their children’s educational needs	275	27	725	67	59	6
Recreational needs	382	35	612	57	85	8
Health-related needs	395	37	601	56	83	7

The deprivation of needs index consists of answers indicating deterioration of possibilities to satisfy one’s needs, and its score ranges from 0 to 8. The average on the scale was 2.1, SD = 2.1.

#### 2.3.5. Mindset changes index

The pandemic, apart from a decrease in the sense of social security, has lead to the loss of control over one’s life and events occurring in it (Table 4). The table shows distribution of answers, which depict the cognitive effects of COVID-19. The loss of psychological resources index was constructed by means of summing up the answers indicating a decrease in self-confidence in the fields presented in the table. Scores ranged from 0 to 6. The higher the score, the bigger the loss of psychological resources. The average on the scale was 1.7, SD = 1.6.

**TABLE 4:** The influence of the pandemic on one’s mindset.

	Decreased		Not changed		Increased	
	***N***	**%**	***N***	**%**	***N***	**%**
The sense of stability and certainty	403	37	621	58	55	5
The sense of achieving one’s goals	300	28	721	67	58	5
The sense of stability in one’s workplace	259	24	767	71	53	5
The sense of control over events in one’s life	330	31	673	62	76	7
The sense of being calm and carefree	435	40	575	53	69	7
The sense of security in a relationship or marriage	128	12	871	81	80	7

#### 2.3.6. The sense of threat of becoming infected with COVID-19

The sense of threat of becoming infected with COVID-19 may be a significant factor connected with the creation of stress in the circumstances of the pandemic. By means of an open-ended question, the respondents were asked to estimate the probability of becoming infected with coronavirus on the scale ranging from 0 to 100. Average chances of becoming infected have been estimated to 40.9%, SD = 25.8.

#### 2.3.7. The level of interest in the information on the pandemic

Numerous studies indicate that the symptoms of stress intensify together with an increased interest in the pandemic. Therefore, this variable was measured as well. The scale used to measure the level of interest in the pandemic consists of four points. The score ranges from 0 to 4. The responses covered the range from “I’m not interested at all” to “I’m very interested.” Higher values indicate a higher interest. The average on the scale was 2.9, SD = 0.7.

### 2.4. Psychological variables

#### 2.4.1. The fear of COVID-19 scale

The Fear of COVID-19 Scale (FCV-19S) was developed by Ahorsu et al. (38). The items of the FCV-19S were constructed basing on extensive review of existing scales on fears, expert evaluations, and participant interviews (38). The FCV-19S consists of 10 items. They were constructed with the use of the following expressions: (1) I am most afraid of COVID-19, (2) It makes me uncomfortable to think about COVID-19, (3) My hands become clammy when I think about COVID-19, (4) I am afraid of losing my life because of COVID-19, (5) While watching news and stories about COVID-19 on social media, I become nervous or anxious, (6) I cannot sleep because I’m worrying about getting COVID-19, (7) My heart races or palpitates when I think about getting COVID-19, (8) COVID-19 is almost always lethal, (9) COVID-19 is an unpredictable disease, (10) I am very worried about COVID-19.

The response for each item was recorded according to a five-point Likert scale ranging from 1 (definitely not) to 5 (definitely yes). The overall score of fear (ranging from 10 to 50) was obtained by adding up each item score. The higher the overall score, the greater the fear of COVID-19. Cronbach α = 0.93. The average fear on COVID-19 Scale was 23,7, SD = 9.

#### 2.4.2. Neuroticism

Neuroticism is deemed a crucial stress factor (39). Neuroticism is a personality trait, which is characterized by a tendency to the occurrence of negative emotions, such as depression, anxiety or anger. Individuals with high neuroticism scores perceive the world as threatening, they are quickly distressed, and it is difficult for them to cope with stressful situations (40). The studies into fear of COVID-19 have shown that neuroticism as one of elements of the Big Five has a negative impact on mental health during the COVID-19 pandemic (41).

The scale used to measure neuroticism was constructed according to the model of the Neuroticism Scale of the Eysenck Personality Questionnaire-Revised (EPQ-R) (42). It consists of 13 items. The response for each item was recorded according to a five-point Likert scale ranging from 1 (definitely not) to 5 (definitely yes). Scores on this scale ranged from 13 to 65, with higher scores indicating higher levels of neuroticism. The coefficient alpha in the present study was 0.87. The average (EPQ-R) was 38.0, SD = 8.

#### 2.4.3. Strategies for coping with stress

The construct put forward by Lazarus and Folkman (43) was used to measure the strategies for coping with stress. In order to measure the strategies, the scale used in longitudinal studies in Poland was applied (44). Dominant strategies were distinguished by means of factorial analysis. The first, problem-focused strategy covered the following responses: (1) I ask other people for help and advice; (2) I get mobilized and do my best to protect myself from it; (3) I comfort myself with a thought that it could have been worse, but at the moment I am healthy; (4) I pray to God for help; (5) I focus on different things which divert my attention and improve my mood. The emotion-focused strategy was indicated by the following responses: (1) I use alcohol, drugs, other psychoactive substances; (2) I give up, don’t know what to do and what to expect; (3) I take sedatives.

#### 2.4.4. Psychological wellbeing

In order to measure wellbeing, an ordinal scale consisting of five items was used. Its value ranges from very dissatisfied (1) to very satisfied (5). The average value for the researched sample was M = 3.71, SD = 1.03.

## 3. Results

### 3.1. Psychological distress

In order to measure this dependent variable (Table 5), the Kessler Psychological Distress Scale (K10) which measures the symptoms of anxiety and depression in the society was used (45). The scale consists of 10 items and its Cronbach α = 0.948. The response for each item was recorded according to a five-point Likert scale ranging from 1 (All of the time) to 5 (None of the time).

**TABLE 5:** The distribution of answers on the distress scale.

	Mean (95% CI)^1^
In the past 4 weeks, about how often did you feel tired out for no good reason?	2.57 (2.51–2.63)
In the past 4 weeks, about how often did you feel nervous?	2.61 (2.55–2.66)
In the past 4 weeks, about how often did you feel so nervous that nothing could calm you down?	2.04 (1.98–2.09)
In the past 4 weeks, about how often did you feel hopeless?	2.33 (2.27–2.39)
In the past 4 weeks, about how often did you feel restless or fidgety?	2.55 (2.49–2.60)
In the past 4 weeks, about how often did you feel so restless you could not sit still?	2.02 (1.97–2.08)
In the past 4 weeks, about how often did you feel depressed?	2.45 (2.39–2.51)
In the past 4 weeks, about how often did you feel that everything was an effort?	2.21 (2.15–2.27)
In the past 4 weeks, about how often did you feel so sad that nothing could cheer you up?	2.11 (2.05–2.17)
In the past 4 weeks, about how often did you feel worthless?	2.10 (2.03–2.16)

^1^CI, confidence interval.

The minimum value on the scale was 10, whereas the maximum value was 50. The average value for the researched sample was M = 22.9, SD = 8.1. According to the scale’s diagnostic criteria [Kessler et al. (45)], the lack of mental disorders (up to 10 points) was observed among 36% of respondents. Mild disorders (20–24 points) were observed among 23% of respondents. An average level of disorders (25–30 points) was observed among 18% of respondents. High levels of disorders (more than 30 points) was observed among 23% of Poles.

The correlation between the scale of stress and the risk factors taken into account in the analysis is presented in Table 6. The results of the correlation analysis indicate that all the three groups of variables are statistically relevant. The variables, such as neuroticism, the fear of COVID-19, low psychological wellbeing, negative strategies, the sense of threat of COVID-19 and evaluation of one’s financial situation are correlated with stress to the most significant extent. This confirms the hypothesis indicating the increase of the pandemic-related stress as a result of the loss of resources. The loss of financial resources, a decrease in social security and a decrease in the sense of psychological security have impact on the creation of stress. The higher the loss of resources, the stronger the psychological discomfort.

**TABLE 6:** Correlation coefficients among all predictors.

Variables	2	3	4	5	6	7	8	9	10	11	12	13	14	15	16	17	18	19	20
1. Gender	-0.03	0	0.03	0.06*	0.02	0.04	0.02	0.05	-0.11**	0.01	0.01	-0.12**	-0.12**	-0.12**	-0.03	-0.14**	0.02	0.01	0.05
2. Age	-	0.20**	0.30**	0.02	-0.12**	0.02	0.20**	0.03	0.09**	0.12**	-0.12**	0.20**	0.15**	-0.28**	0.14**	0.19**	-0.13**	0.10**	-0.23**
3. Place of residence	-	-	0.13**	0.02	-0.06**	0.07**	0.08**	0	0.07**	0.07**	0.01	0.10**	0.06**	-0.08**	0.02	0.04	0	0.02	-0.04
4. Education	-	-	-	0.11**	-0.02	-0.02	0.11**	0.04	0.13	0.02	0.17**	0.15**	0.20**	-0.13	-0.03	0.09**	-0.04	0.1	-0.11
5. Evaluation of financial status	-	-	-	-	0.18**	-0.06	0.02	0.04	-0.19**	-0.22**	-0.08**	-0.17**	0.03	-0.27**	-0.17**	0	0.14**	-0.35**	0.25**
6. Social capital index	-	-	-	-	-	-0.08**	0	0.03	-0.16**	-0.11**	0.01	-0.09**	0	-0.11**	-0.06*	0.01	-0.07	0.23**	-0.12**
7. Religiosity	-	-	-	-	-	-	-0.07**	-0.01	0.08**	0.01	0	0.09**	-0.01	-0.09**	-0.06*	-0.16**	0.01	-0.12**	0.01
8. The level of interest in the information on the COVID-19 pandemic	-	-	-	-	-	-	-	0.28**	0.17**	0.08**	0.09**	0.13**	0.26**	0.07	0.42**	0.33**	0.06*	0.01	0.12**
9. The sense of threat of becoming infected with COVID-19	-	-	-	-	-	-	-	-	0.13**	0.03	0.15**	0.12**	0.19**	0.19**	0.20**	0.16**	0.11**	0.02	0.29**
10. Mindset change index	-	-	-	-	-	-	-	-	-	0.29**	0.19**	0.55**	0.29**	0.18**	0.20**	0.27**	0.12**	-0.23**	0.16**
11. Life position decrease index	-	-	-	-	-	-	-	-	-	-	0.09**	0.27**	0.18**	0.08**	0.15**	0.12**	0.08**	-0.21**	0.12**
12. Loss of economic resources index	-	-	-	-	-	-	-	-	-	-	-	0.20**	0.18**	0.09**	0.09**	0.14**	0.15**	-0.03	0.14**
13. Deprivation of needs index	-	-	-	-	-	-	-	-	-	-	-	-	0.31**	0.02	0.08**	0.25**	0.05	-0.11**	0.05
14. Life changes index	-	-	-	-	-	-	-	-	-	-	-	-	-	0.05	0.25**	0.42**	0.06	-0.01	0.09**
15. Neuroticism	-	-	-	-	-	-	-	-	-	-	-	-	-	-	0.35**	0.09*	0.27**	-0.42**	0.76**
16. FCV-19S	-	-	-	-	-	-	-	-	-	-	-	-	-	-	-	0.35**	0.21**	-0.08*	0.46**
17. Problem-focused strategy	-	-	-	-	-	-	-	-	-	-	-	-	-	-	-	-	0	0.09**	0.09**
18. Emotion-focused strategy	-	-	-	-	-	-	-	-	-	-	-	-	-	-	-	-	-	-0.19**	0.32**
19. Wellbeing	-	-	-	-	-	-	-	-	-	-	-	-	-	-	-	-	-	-	-0.39**
20. Psychological Distress K10	-	-	-	-	-	-	-	-	-	-	-	-	-	-	-	-	-	-	-

*p < 0.05; **p < 0.01.

The hierarchical regression analysis (Table 7) was used to study the relationship between socio-demographic, psychological, social effect variables and the level of stress. In the first stage, socio-demographic variables were introduced, in the second stage, the variables measuring negative effects of lockdown and the level of interest in the information on the pandemic were applied, and in the third stage, the psychological variables were introduced to the model.

**TABLE 7:** Results of multiple regression analyses predicting the level of psychological distress.

Variables	Model I		Model II		Model III	
	**β**	***P***	**β**	***P***	**β**	***P***
Gender	-0.040	0.161	-0.048	0.076	0.019	0.303
Age	-0.240	0.000	-0.261	0.000	-0.085	0.052
Place of residence	0.009	0.753	0.001	0.959	0.015	0.424
Education	0.030	0.315	0.064	0.029	-0.005	0.811
Evaluation of financial status	0.227	0.000	0.176	0.000	-0.009	0.063
Social capital index	-0.108	0.000	-0.094	0.001	-0.025	0.190
Religiosity	-0.002	0.938	0.010	0.726	0.067	0.000
The level of interest in the information on the COVID-19 pandemic	-	-	0.089	0.003	-0.008	0.686
The sense of threat of becoming infected with COVID-19	-	-	0.250	0.000	0.068	0.001
Loss of psychological resources index	-	-	0.091	0.007	-0.016	0.488
Life position decrease index	-	-	0.075	0.010	0.030	0.123
Loss of economic resources index	-	-	0.036	0.216	0.039	0.046
Deprivation of needs index	-	-	-0.068	0.045	0.011	0.421
Life changes index	-	-	-0.035	0.247	-0.006	0.764
Neuroticism	-	-	-	-	0.614	0.000
FCV-19S	-	-	-	-	0.231	0.000
Problem-focused strategy	-	-	-	-	-0.029	0.186
Emotion-focused strategy	-	-	-	-	0.069	0.000
Wellbeing	-	-	-	-	-0.060	0.003
F (*p* ≤ 0.000)	23.575		24.460		107.128	
R square	0.134		0.243		0.658	
Standard error	7.642		7.164		4.830	

The results of the hierarchical regression analysis indicate the fact that demographic variables explain 13% of stress variance. Higher levels of stress were experienced by young people, lonely people, people deprived of social support and assessing their financial standing as worse.

Introducing variables measuring social effects of lockdown to the model resulted in a significant change in corrected R2, [delta corrected R2 = 0.110; F change (7,1074) = 22,094, *p* < 0.000], and indicates that introducing those variables has increased the level of explained variance to 24%. The sense of threat of becoming infected with COVID-19, the level of interest in the pandemic, the loss of psychological resources, a decrease in one’s life position and deprivation of needs turned out to be statistically relevant. The variables from the first model (age, education, evaluation of one’s financial standing, and social capital) have retained their impact. In the third stage, upon introducing psychological variables, the level of explained variance was 66%. The change in the explained variance was statistically relevant. A significant change in corrected R2, [delta corrected R2 = 0.414; F change (5,1059) = 256.401, *p* < 0.000] was observed. In this model, neuroticism was the strongest stress predictor. The FCV-19S scales had a lesser impact on stress levels. Negative strategies and wellbeing had a lesser influence on stress. Age, religiousness from the first model remained statistically relevant. The fear of becoming infected and the loss of economic resources from the first model were introduced in the calculation.

## 4. Discussion

The results of research confirm the general assumption that the COVID-19 pandemic and its consequences will negatively affect the Polish society. Stress will be determined by the loss of financial, social and psychological resources (28).

The results of linear regression analysis partially confirm the assumed hypothesis. By means of analyses, it has been shown that three factors determine the level of stress. Socio-demographic variables explain only 13% of the dependent variable variance. Higher levels of stress were observed among young people, and people who were devoid of social capital and evaluated their financial standing as worse.

The predictors distinguished in the first regression model indicate that the highest level of distress could be observed among the youngest age groups. These results are consistent with the findings of researchers from many different countries (11, 46). Meta-analyses also indicate a worse mental condition of young people in the time of the COVID-19 pandemic (47). Higher levels of stress may be explained by the fact that, first of all, just before the pandemic youth had a worse mental condition than adults (48, 49). The pandemic might have intensified these trends. Secondly, among younger age groups, peer relations are one of the main needs (50). Quarantine and social distancing have resulted in a deprivation of this need, at the same time deepening the distress (51). A higher distress was observed among people with lower social capital, which may indicate the crucial role of social support in the time of the threat of the pandemic. Married people and people who have others willing to help facilitated coping with the pandemic, which is indicated by many other studies (52). Respondents in a worse financial situation had a worse mental health. This may mean that the lack of financial resources lowers adaptive capabilities.

In the second model of the regression analysis, the indices aimed at indicating secondary effects of the pandemic, such as limitations of social activities or limitations in satisfying one’s needs were introduced. It has been shown that the level of explained variance of the distress scale has increased by more than 10%. Variables, such as age, evaluation of one’s financial situation and social capital turned out to be statistically relevant. Moreover, it was observed that people indicating a higher interest in the pandemic, which is presented in the media are more stressed. It is consistent with the results of research indicating a negative influence of exposure to media on mental health during the COVID-19 pandemic (53, 54).

The probability of becoming infected with coronavirus, a decrease in one’s life position and experiencing financial losses due to the pandemic is correlated with higher levels of stress. This may confirm the theory of the impact of the loss of resources on experienced stress (27). A prospective loss of health resources and the actual loss of financial resources increase the results on the stress scale.

In the third model, psychological variables were introduced to the analysis, thanks to which the level of explained variance on the stress scale has increased to 40%. Furthermore, significant predictors of the experienced stress have changed as well. Age has retained its impact on the level of stress. Religiousness pertaining to coronavirus has been included in the explanation of the dependent variable. Higher levels of stress were observed among people undecided in terms of faith. Faith, being a member of a religious community may provide support and decrease stress levels, which is confirmed by research (55). Religion helps believers with handling stress (56, 57). There are also results of research indicating that during the pandemic, a correlation between religiousness and stress levels was observed (58). This may mean that the media have a negative impact on mental health of individuals, which is confirmed by other studies as well (59, 60). Nonetheless, the results on the scale of fear of COVID-19, emotion-focused strategies, the lack of psychological wellbeing and the neuroticism scale had the strongest impact on the observed stress levels. A high level of fear of COVID-19 and emotion-focused strategies were correlated with higher levels of stress. This relationship has been observed in other studies as well (61, 62). Nevertheless, among all of the variables, neuroticism had the strongest force of impact. This may mean that neuroticism is a moderator of stress and acts as an intermediary in the perception of reality. In the situation of threat of the pandemic, it increases the sense of threat and by the same means it significantly increases the level of psychological distress, which is indicated by other studies (23, 63, 64).

## 5. Conclusion

The results of research conducted after the first wave of the COVID-19 pandemic (7 months after the occurrence of COVID-19 in Poland) indicate that only 36% of Poles have not experienced psychological distress. The respondents experienced anxiety, irritability, fatigue and gloom most often. The pandemic and threat posed by it which last for a long time have led to a bad mental condition.

The main factor which had impact on psychological distress was neuroticism. Emotionally unstable individuals, people with higher levels of fear and depression had a difficulty coping with the threat and the situation of uncertainty in the time of the pandemic. Apart from neuroticism, socio-demographic variables and experienced financial losses had impact on distress. The results of research indicate that psychological, social and financial resources may protect individuals from the COVID-19-related distress. Therefore, during and after the pandemic, the main focus should be providing people who are lonely, who lost their jobs and income due to the pandemic and those who present neurotic disorders with support and care.

## 6. Limitations

The presented research has certain limitations. First of all, research was conducted during a relative decrease in the number of COVID-19 incidences, after the summer break, during which the society could have forgotten about the threat, which as a result could influence the obtained results. Secondly, the on-line survey (CAWI) has an over-representation of respondents with education higher than Poles in general, in spite of all the attempts to provide a representative sample. Elderly and poor people as well as those with no Internet connection could have been passed over in the research sample. Thirdly, the study was of a cross-cutting nature. Therefore, it is difficult to evaluate to what extent the COVID-19 influenced the mental health of Polish society.

## Data availability statement

The raw data supporting the conclusions of this article will be made available by the authors, without undue reservation.

## Ethics statement

The studies involving human participants were reviewed and approved by ethical approval: All procedures were conducted in accordance with the ethical standards of the institutional and/or Polish National Research Committees, and with the 1964 Helsinki declaration and its later amendments or comparable ethical standards. The Ethics Board of the Pedagogical University of Kraków approved the studies. Informed consent was obtained individually from all participants in volvedin the studies. The studies were conducted using the online Polish ARIADNA participant panel, which has over 110,000 active adult panel members. E-mail invitations were sent to potential participants diversified in terms of their age, gender, and level of education. As compensation for their involvement, participants were awarded points that were redeemable for rewards from a pool of several hundred products offered by the platform running the panel. Parental consent was obtained for study participants aged 15–18. The patients/participants provided their written informed consent to participate in this study.

## Author contributions

The author confirms being the sole contributor of this work and has approved it for publication.
